# Modulation of Ca^2+^ Signals by Epigallocatechin-3-gallate(EGCG) in Cultured Rat Hippocampal Neurons

**DOI:** 10.3390/ijms12010742

**Published:** 2011-01-20

**Authors:** Jiang-Hua Wang, Jin Cheng, Cai-Rong Li, Mao Ye, Zhe Ma, Fei Cai

**Affiliations:** 1 College of Fisheries, Huazhong Agricultural University, Wuhan 430070, Hubei, China; E-Mail: whtjwjh@163.com; 2 Department of Pharmacy, The Affiliated Xiangfan Hospital of TongJi Medical College of Huazhong University of Science & Technology, Xiangfan 441021, Hubei, China; E-Mail: 6522526@qq.com; 3 Department of Pharmacology, Medical College, Xianning University, Xianning 437100, Hubei, China; E-Mails: 12368184@qq.com (C.-R.L.); 156307261@qq.com (Z.M.); 4 Department of Osteopaedics, Center Hospital of Xianning, Xianning 437100, Hubei, China; E-Mail: 10255877@qq.com

**Keywords:** EGCG, calcium imaging, intracellular Ca^2+^, phopholipase C, hippocampal neuron

## Abstract

Green tea has been receiving considerable attention as a possible neuroprotective agent against neurodegenerative disease. Epigallocatechin-3-gallate (EGCG) is the major compound of green tea. Calcium signaling has profound effects on almost all aspects of neuronal function. Using digital calcium imaging and patch-clamp technique, we determined the effects of EGCG on Ca^2+^ signals in hippocampal neurons. The results indicated that EGCG caused a dose-dependent increase in intracellular Ca^2+^ ([Ca^2+^]_i_). This [Ca^2+^]_i_ increase was blocked by depleting intracellular Ca^2+^ stores with the endoplasmic reticulum Ca^2+^ pump inhibitor thapsigargin and cyclopiazonic acid. Furthermore, EGCG-stimulated increase in [Ca^2+^]_i_ was abolished following treatment with a PLC inhibitor. However, EGCG inhibited high-voltage activated Ca^2+^ currents (I_HVA_) and NMDA-induced inward currents (I_NMDA_). These data suggest that EGCG triggers a cascade of events: it activates phospholipase C (PLC), mobilizes intracellular Ca^2+^ stores, raises the cytosolic Ca^2+^ levels, and inhibits the VGCC and NMDA receptors-mediated Ca^2+^ influx through a process that remains to be determined.

## 1. Introduction

Green tea polyphenols are natural plant flavonoids and comprise many types of catechin. Among them, (–)-epigallocatechin gallate (EGCG) is the major polyphenol component and primarily responsible for the green tea effect. Previous studies on the biological activities of EGCG were mostly focused on its beneficial effects, including antioxidant [1,2], anticarcinogenic [3–5], and anti-inflammatory properties [6]. Also, EGCG can penetrate the brain’s blood barrier [7] and has neuroprotective effects.

Calcium (Ca^2+^) is a ubiquitous intracellular signal responsible for controlling numerous cellular processes, such as fertilization, proliferation, development, learning and memory [8]. Maintenance of intracellular Ca^2+^ homeostasis is crucial for cell survival. The site, magnitude, and kinetics of Ca^2+^ changes determine the biological consequences of Ca^2+^ signaling in the neurons [9–11]. One biological action attributed to EGCG is the ability to influence intracellular Ca^2+^([Ca^2+^]_i_) in both non-excitable and excitable cells. EGCG reportedly attenuates the a-amino-3-hydroxy-5-methyl-4-isoxazolepropionic acid (AMPA)-induced increase in [Ca^2+^]_I_ in hippocampal neurons [12]. In addition, EGCG also reduced both *N*-methyl-4-phenyl-1,2,3,6-tetrahydropyridine (NMDA) and kainate-induced [Ca^2+^]_i_ increase by attenuating both ionotropic Ca^2+^ influx and Ca^2+^-induced Ca^2+^ release (CICR) in PC12 cells, but it did not affect matabotropic receptor-mediated Ca^2+^ release [13]. These studies have focused to elucidate the protective effects of EGCG after the treatment of cells with cytotoxic agents. Recently, it has been demonstrated that EGCG mediated stimulation of [Ca^2+^]_i_ in cultures of rat hippocampal neurons is partially responsible for the death of hippocampal neurons induced by EGCG [14]. Excessive elevation of [Ca^2+^]_i_ produces deleterious effects and results in cell death. Thus, it is essential for cells to carefully buffer intracellular calcium and to precisely regulate calcium entry [15]. We hypothesized that EGCG at low concentrations could result in a relatively small increase in [Ca^2+^]_i_, which would be compensated for by the calcium-buffering mechanisms of cells. However, high-concentration EGCG produced excessive elevation of [Ca^2+^]_i_ which was possible beyond the buffering capacity of neurons and led to a cascade of cellular pathological events [14].

Currently, the detailed mechanisms by which EGCG regulated [Ca^2+^]_i_ in neurons remain unclear. The present study characterized EGCG-regulated Ca^2+^ signaling by employing calcium imaging and whole cell patch-clamp techniques. The results indicated that EGCG increased [Ca^2+^]_i_ in hippocampal neurons in a dose-dependent manner. The regulation, which was mediated by intracellular stores mobilization via activation of a PLC-IP3 pathway, led to suppression of high-voltage activated Ca^2+^ currents (I_HVA_) and NMDA-induced inward currents (I_NMDA_). These data shed new light on the understanding of pharmacological action of EGCG in hippocampal neurons.

## 2. Results and Discussion

### 2.1. EGCG Elevates [Ca^2+^]_i_ in Rat Hippocampal Neurons in a Dose-Dependent Manner

**Figure 1:**
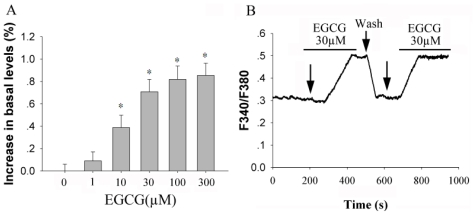
EGCG induced increase of intracellular calcium levels in hippocampal neurons. (**A**) Summary data of increases in [Ca^2+^]_i_ for each dose of EGCG from three independent experiments. [Ca^2+^]_i_ increase was recorded as described in the Experimental section and expressed as [(F340/F380)_S_-F340/F380)_B_]/(F340/F380)_B_. * *P* < 0.05 *vs*. corresponding value in control cultures; (**B**) Hippocampal neurons were treated with 30 μM EGCG for the designated time. The arrows indicate the time of EGCG application or washout.

In the first set of experiments, we employed the [Ca^2+^]_i_ imaging technique to study the dynamic alteration of intracellular calcium mediated by EGCG in primary culture of rat hippocampal neurons. From the results, the effect of EGCG was significant at 10 μM (*P* < 0.05, *n* = 29), and reached a maximal level at a concentration of 100 μM (Figure 1B). The EC^50^ for potentiation effect was 11.6 ± 1.6 μM (fitted with Sigmaplot version 9.0 to the Hill equation). Calcium mobilization was initially detected 100 s following the application of 30 μM EGCG and rapidly reached a plateau in 236 ± 48 s. Repeated 30 μM EGCG stimulation produced equal response and this response was fully reversed after washout with ACSF. The response were detected in 59% (34 of 58 cells tested) of neurons, resulting in a 70.8 ± 11.0% increase over basal levels.

### 2.2. EGCG-Evoked Enhancement of [Ca^2+^]_i_ Depends on the Release from Intracellular Calcium Store and Ca^2+^ Influx

**Figure 2:**
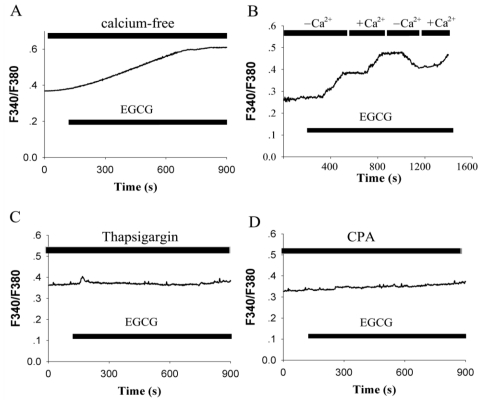
EGCG-mediated increase in [Ca^2+^]_i_ depends on release of Ca^2+^ from intracellular calcium stores and Ca^2+^ influx. (**A**) Superfusion of cultured neurons was switched to a Ca^2+^-free solution when 30 μM EGCG was added; (**B**) [Ca^2+^]_i_ increase was halted while Ca^2+^-free solution was applied and restored after switching back to Ca^2+^-containing solution during EGCG-induced increase in [Ca^2+^]_i_. +Ca^2+^, Ca^2+^-containing ACSF applied. –Ca, Ca^2+^-free ACSF applied; (**C**) Application of 1 μM thapsigargin to delete the intracellular calcium store completely blocked EGCG-induced [Ca^2+^]_i_ increase in hippocampal neurons; (**D**) Application of 10 μM CPA to deplete the intracellular calcium store, EGCG failed to evoke a significant increase in [Ca^2+^]_i_. The experiments were repeated at least three times and representative data are shown for each treatment.

To explore the respective contribution of Ca^2+^ influx from the extracellular compartment or the release from intracellular Ca^2+^ stores on EGCG-induced [Ca^2+^]_i_ elevation in hippocampal neurons, the standard extracellular solution was replaced by Ca^2+^-free solution. In the absence of extracellular Ca^2+^, 30 μM EGCG was able to induce a significant increase of basal [Ca^2+^]_i_ (54 ± 8.1% over unstimulated level, *n* = 28 ), although the magnitude of elevation (Figure 2A) was less than that in the presence of extracellular Ca^2+^ (Figure 2B). This suggests that calcium release from intracellular stores was partly responsible for the induced Ca^2+^ elevation. As shown in Figure 2B, EGCG induced Ca^2+^ increase was blunt while Ca^2+^-free medium was applied, but recovered again after reperfusion with Ca^2+^-containing medium.

The importance of Ca^2+^ release from intracellular stores in triggering the elevation of [Ca^2+^]_i_ was further investigated. After pre-incubation with 1 μM thapsigargin to deplete the intracellular calcium store in neurons bathed in Ca^2+^-containing medium, EGCG failed to evoke a significant increase in [Ca^2+^]_i_ (*n* = 31, Figure 2C). Similar results were obtained when cyclopiazonic acid (CPA), an inhibitor of sarcoplasmic/endoplasmic reticulum Ca^2+^-ATPase, was applied (*n* = 27, Figure 2D). These experiments indicated that Ca^2+^ release from intracellular stores was necessary and sufficient for EGCG-induced [Ca^2+^]_i_ rise.

### 2.3. Activation of Phospholipase C (PLC) Signaling Pathways is Essential for EGCG-Stimulated [Ca^2+^]_i_ Elevation

**Figure 3:**
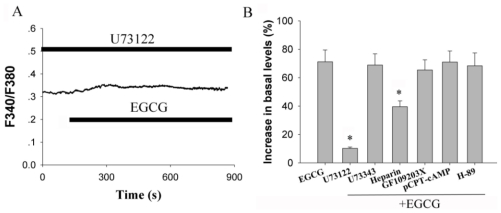
Activation of phospholipase C (PLC) signaling pathways mediates EGCG-induced [Ca^2+^]_i_ elevation. (**A**) Pretreatment with 10 μM U73122 blocked EGCG-induced [Ca^2+^]_i_ increase in hippocampal neurons (*n* = 20). Experiments were repeated at least 3 times; (**B**) Summary data of the results with U73122, U73343 (10 μM), heparin (20 mg/mL), GF109203X (2 μM), p-CPT-cAMP (200 μM) and H-89 (30 μM) preincubation on EGCG-mediated elevation in hippocampal neurons. * *P <* 0.05 compared with EGCG alone.

To ascertain which intracellular signaling pathway is activated in EGCG-induced Ca^2+^ release from intracellular calcium stores, cells were treated with 10 μM U73122, a PLC inhibitor. Notably, the increase in intracellular calcium levels by EGCG was prevented by pre-treatment with U73122 (*n* = 20, Figure 3A), whereas U73343, an inactive analog of U73122 (without inhibitory activity), did not alter EGCG-stimulated [Ca^2+^]_i_ elevation (*n* = 21, *P* > 0.05).

Activation of PLC is known to stimulated PI hydrolysis, IP^3^ and 1,2-diacylglycerol (DAG) production. Subsequently, IP_3_ stimulates the Ca^2+^ release from endoplasmic reticulum (ER) stores; DAG leads to activation of protein kinase C (PKC). To test which pathway was involved in EGCG-mediated elevation of [Ca^2+^]_i_, primary cultured hippocampal neurons were pretreated with heparin (20 mg/mL, a competitive antagonist of IP_3_), EGCG-evoked responses were attenuated to 39.6 ± 4.1% over basal levels (*n* = 12, *P* < 0.05 *vs.* EGCG alone). When cells were treated with PKC inhibitor GF109203X (2 μM), the stimulation effects of EGCG were not noticeably altered. The results suggested that the potentiation effect of EGCG was partially dependent on the activation of IP_3_. To determine whether coupling to the AC signaling pathway was affected by EGCG, cells treated with pCPT-cAMP, a membrane permeable cAMP analog, was examined. In contrast, pretreatment with 200 μM pCPT-cAMP for 3 min had no effect on the increase in EGCG-mediated intracellular calcium levels (*n* = 19, *P* > 0.05). Meanwhile, H-89, a permeable PKA inhibitor, did not alter EGCG-stimulated elevation (*n* = 23, *P* > 0.05). These data suggested that EGCG mediated calcium signaling was not dependent on PKA pathway, but a PLC dependent pathway.

### 2.4. Voltage-Gated Calcium Channel and Receptor-Operated Calcium Channel Contribute to the Increase of [Ca^2+^]_i_

**Figure 4:**
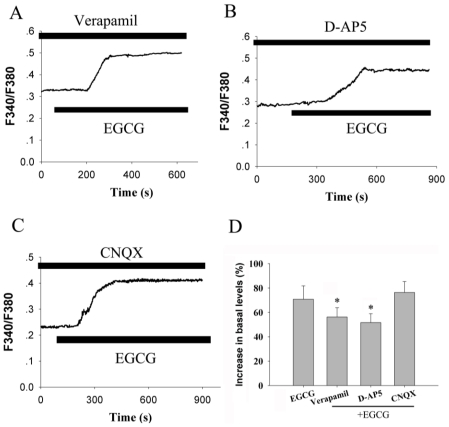
Voltage-gated calcium channels and receptor-operated calcium channels contribute to EGCG-induced Ca^2+^ influx. (**A**) Application of 10 μM verapamil for 3 min prior to the addition of 30 μM EGCG reduced the drug-stimulated [Ca^2+^]_i_ elevation (*n =* 21); (**B**) Pretreatment with 50 μM D-AP5 for 3 min before the addition of 30 μM EGCG reduced the drug-stimulated [Ca^2+^]_i_ elevation (*n =* 19); (**C**). 30 μM CNQX failed to have an effect on EGCG-stimulated [Ca^2+^]_i_ elevation (*n =* 23); (**D**) Summary data of the results with verapamil, D-AP5 and CNQX preincubation on EGCG-mediated elevation in hippocampal neurons. *P < 0.05 compared with EGCG alone.

Although we demonstrated that Ca^2+^ influx from the extracellular compartment contributed to the EGCG induced [Ca^2+^]_i_ elevation, the exact pathway involved in this regulation is unclear. To address this issue, we first employed verapamil to block voltage-gated calcium channels (VGCC). As shown in Figure 4A, 10 μM verapamil prevented the potentiating effect of 30 μM EGCG on intracellular calcium levels from 70.8 ± 11.0% to 56.3 ± 7.5% (*n =* 21, *P <* 0.05).

Next, the role of receptor-operated calcium channels (ROCC) in this process was checked. As depicted in Figure 4B, 50 μM DL-2-amino-5 phonovaleric acid (D-AP5) attenuated the EGCG-induced elevation of [Ca^2+^]_i_. The maximal increases in [Ca^2+^]_i_ over basal levels were 51.7 ± 7.2% (*n =* 19, *P <* 0.05). However, 10 μM 6-cyano-7-nitroquinoxaline-2,3-dione (CNQX), a selective AMPA/kainite receptor competitive antagonist, did not produce any inhibition on EGCG induced elevation of free calcium in the neurons (Figure 4C). This result indicated that activation of the NMDA receptor contributed to the calcium influx stimulated by EGCG in hippocampal neurons.

### 2.5. EGCG Suppresses I_HVA_ and I_NMDA_ via Elevating Intracellular Ca^2+^ Concentration

**Figure 5:**
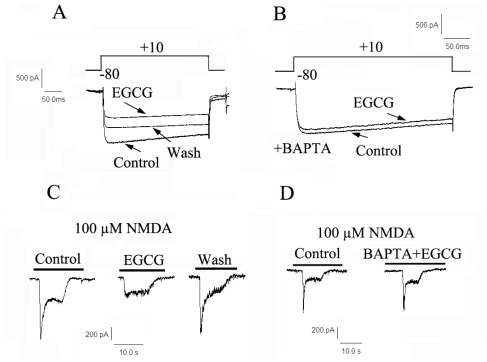
The EGCG-induced inhibitory effect on I_HVA_ and I_NMDA_ are Ca^2+^-dependent. (**A**) 30 μM EGCG reversibly decreased high-voltage-activated (HVA) Ca^2+^ currents in primary cultured hippocampal neurons. Voltage protocol is shown on the top; (**B**) Representative trace showing dialysis with 15 mM bis-(*o*-aminophenoxy)-*N*,*N*,*N*’,*N*’-tetraacetic acid (BAPTA) significantly reduced the inhibitory modulation of EGCG on I_HVA_; (**C**) 30 μM EGCG reversibly decreased NMDA activated currents in primary cultured hippocampal neurons; (**D**) Representative traces showing dialysis with 15 mM BAPTA significantly attenuated the inhibitory effects of EGCG.

To further determine the roles of VGCC and NMDA receptor in the EGCG induced [Ca^2+^]_i_ elevation, the whole cell patch-clamp technique was employed to study the effects of EGCG on high-voltage-activated calcium currents (I_HVA_) and NMDA-induced inward currents (I_NMDA_). I_HVA_ were elicited by a depolarization from holding potential of −80 mV to +10 mV. EGCG 30 μM caused a reduction in I_HVA_ from 63.2 ± 5.9 pA.pF^−1^ to 46.7 ± 5.3 pA.pF^−1^(*n =* 21, *P <* 0.05). I_HVA_ decreased by EGCG was irreversible after EGCG was washed out (Figure 5A). The cells were further dialyzed with high concentration of fast Ca^2+^ chelator BAPTA (15 mM), which is sufficient to maintain [Ca^2+^]_i_ at a nanomolar level. As shown in Figure 5B, BAPTA almost abolished EGCG-induced inhibitory effect completely (*n =* 8, *P <* 0.05 *vs*. EGCG alone). These results revealed that the inhibition induced by EGCG was Ca^2+^-dependent.

NMDA (100 μM), an agonist of NMDA receptors, generated inward whole-cell membrane currents (I_NMDA_) at a holding potential of −50 mV in cultured hippocampal neurons. The currents were blocked by the NMDA antagonist D-AP5, but not by the non-NMDA glutamate receptor antagonist CNQX (data not shown). As depicted in Figure 5C, with treatment with EGCG (30 μM) for 3 min, the amplitude of I_NMDA_ was decreased by 62.9 ± 6.1% (*n =* 10, *P <* 0.05), whereas the inhibitory effects of EGCG on I_NMDA_ were blocked by addition of BAPTA (*n =* 9, Figure 5D).

### 2.6. Discussion

In the present study, we found that EGCG elevated [Ca^2+^]_i_ in a dose-dependent manner in cultured hippocampal neurons, which was initially driven by Ca^2+^ release from intracellular stores. Our data further demonstrated that EGCG induced inhibition of I_HVA_ and I_NMDA_ was mediated by elevation of intracellular calcium through activation of a PLC-IP3 pathway. This biochemical cascade induced by EGCG is thought to be causative for the neuroprotective/neurotoxic effects of EGCG exposure.

EGCG, as the main catechin polyphenols of tea, has been investigated experimentally. Attention has already been paid to the effects of EGCG on the nervous system in previous investigations. It has been reported that EGCG exerts neuroprotective effects in various models of toxicity induced by ischemia, glutamate, *N*-methyl-4-phenyl-1,2,3,6-tetrahydropyridine(MPTP), oxidative stress and Aβ peptides [13,16–18]. In contrast to the neuroprotective effects of the EGCG, increasing evidence indicates that there is a concentration-dependent window of pharmacological action, in which, at high concentrations, EGCG has pro-oxidant/pro-apoptotic activity [19,20]. EGCG showed some maternal toxicity and reduced the growth rate of offspring in company with a slight increase in pup loss at high dose in genotoxity and teratogenicity studies [21,22]. And EGCG increased DNA strand breakage in purified blood lymphocytes at high doses [23]. More similarly, EGCG was reported to decrease the neuronal activity of medial vestibular nuclear neurons by playing a role in decreasing the neuronal activity of contralateral vestibular nuclei [24]. More recently, EGCG was found to block the voltage-gated sodium channel currents (I_Na_) at the concentration of 400 μM and higher in hippocampal neurons [25].

[Ca^2+^]_i_. is central to multiple signal transduction pathways to accomplish a variety of biological functions. Our result is in agreement with a previous report that EGCG could cause a [Ca^2+^]_i_ elevation in a dose-dependent way [14]. Two major intracellular sources contribute to [Ca^2+^]_i_ mobilization: an intracellular release through plasma membrane channels and a internal reservoir in the ER and mitochondria [26]. Our data showed that EGCG could still induce an increase in [Ca^2+^]_i_ in the absence of extracellular Ca^2+^ and was abolished in cells that were depleted of intracellular calcium stores by thapsigargin and CPA, which suggested that the mobilization of Ca^2+^ in the ER was necessary and sufficient for this process. However, inhibition of EGCG-induced [Ca^2+^]_i_ increase by removal of extracellular Ca^2+^ recovered again after reperfusion with Ca^2+^-containing medium. These results suggest that Ca^2+^ influx is partly involved in the EGCG induced calcium elevation. In our present study, a large proportion of EGCG-induced elevation of [Ca^2+^]_i_ was still apparent in the presence of VGCC and ROCC blockers, which suggested that these entry pathways did not represent the principal mode of elevation of [Ca^2+^]_i_. Verapamil and D-AP5 slightly reduced EGCG-induced increase in [Ca^2+^]_i_ in hippocampal neurons. However, EGCG inhibited I_HVA_ and I_NMDA_, suggesting that the EGCG-induced Ca^2+^ influx occurred mainly though other pathways. Capacitative calcium entry (CCE) via store-operated calcium channel is considered to be the major mechanism for influx of Ca^2+^ in a nonexcitable cell system. It is generally believed that CCE operation is nonexistent in neurons, although there were some disagreements [27]. Although our data did not find the occurrence of CCE in cultured hippocampal neurons, this pathway could not be excluded to be involved in EGCG-induced Ca^2+^ influx.

Excessive elevation of [Ca^2+^]_i_ would result in intracellular calcium overload, which triggers a cascade of events leading to cell death. Calcium-buffering mechanism is essential for cells to reduce [Ca^2+^]_i_. One target of intracellular calcium is voltage-dependent and ligand-gated ion channels [28]. Intracellular calcium can inactivate these channels by binding directly to ion channels or via Ca^2+^-dependent enzymes, such as calmodulin and calcineurin [29]. Ca^2+^-dependent inactivation of HVA Ca^2+^ currents restricts the entry of Ca^2+^ into the cytoplasm, which is a negative feedback mechanism between Ca^2+^ entry and the intracellular Ca^2+^ concentration [30]. This mechanism was reported to be involved in nicotine-induced neuroprotection [31]. The present study demonstrated that EGCG reversibly inhibited I_HVA_ and I_NMDA_ in primary cultured hippocampal neurons. Chelation of intraculluar Ca^2+^ with BAPTA reduced the effects of EGCG, which suggested that EGCG induced inhibition of I_HVA_ and I_NMDA_ was dependent on Ca^2+^ release from the intracellular stores. We argue that this type of coordinated modulation in signaling processes might protect neurons from Ca^2+^ overload.

EGCG is known to bind to several proteins and affects the activity of enzymes and receptors. It has been reported to act through phosphoinositide 3-kinase (PI 3-kinase), Akt/protein kinase B (Akt/PKB), tyrosine kinases, protein kinase C (PKC), and mitogen activated protein kinase (MAP kinase) signaling cascades [32]. Inhibitory or stimulatory actions at these pathways are likely to influence cellular function profoundly. However, in our experiments, the limb of these signaling cascades did not participate in the EGCG-induced elevation of [Ca^2+^]_i_. A clear understanding of the signal transduction mechanisms is a key to the evaluation of action of EGCG, either as a neuroprotective agent or neurotoxic agent [33]. In our study, inhibitors of PKA and PKC did not alter the effects of EGCG. In contrast, blockade of IP_3_ receptors with heparin or depletion of intracellular calcium store with thapsigargin and CPA disrupted the EGCG-induced increase in [Ca^2+^]_i_. These data indicated that EGCG stimulated [Ca^2+^]_i_ via PLC-IP3 pathway in hippocampal neurons.

## 3. Experimental Section

### 3.1. Materials

EGCG, U-73122,U-73343, p-CPT-cAMP, CPA, cyclopiazonic acid, NMDA, H-89, DL-2-amino-5-phos-phonovaleric acid (AP-5), 6-cyano-7-nitroquinoxaline-2,3-dione (CNQX), heparin, GF109203X, verapamil, and thapsigargin were purchased from Sigma (St. Louis, MO, USA). Fura-2/AM was obtained from Biotium (Hayward, CA, USA). DMEM/F12 and B27 supplement were obtained from Gibco Invitrogen Corporation (Carlsbad, CA, USA). Other general agents were available commercially. Other agents were purchased from commercial suppliers. Agents were prepared as stock solutions with sterile water except difedipine, U-73122, U-73343 and Fura-2/AM, which were dissolved in dimethylsulfoxide (DMSO) and stored at −20 °C. They were diluted to the final concentrations before application. The final concentration of DMSO was <0.05%. No detectable effect of the vehicles was found in our experiments.

### 3.2. Cell Culture

Neonatal SD (Sprague-Dawley) rats (day 0–3) of both sexes were obtained from the Center for Disease Control of Hubei Province, China. All experiments were conducted in accordance with the *National Institutes of Health Guide for the Care and Use of Laboratory Animals*. All experiments conformed to named local guidelines on the ethical use of animals and all efforts were exerted to minimize the number of animals used and their suffering. Neurons were isolated as previously described with some modification [25]. Briefly, hippocampi of newborn rats were dissected and rinsed in ice cold dissection buffer. Blood vessels and white matter were removed and tissues were incubated in 0.125% trypsin for 25 min at 37 °C. Neurons were collected by centrifugation and resuspended in Dulbecco’s modified Eagle’s medium (DMEM) and F-12 supplement (1:1) (Gibco Invitrogen corporation) with 10% fetal bovine serum (heat-inactivated, Hyclone), 2 mM L-glutamine (Sigma), and Penicillin (100 U/mL)-streptomycin (100 U/mL). Cells were plated at a density of 10^4^–10^5^ per 35 mm^2^ on coverslides precoated with poly-L-lysine and kept at a 37 *°*C in a 5% CO_2_ incubator. After 24 h, the culture medium was changed to DMEM medium containing 2% B27 and 2 mmol/L glutamine. Astrocytes were minimized by treating the culture with cytarabine (10 μM) on day 3. The medium was replaced with fresh medium every 3 days. Experiments were performed on day 5–7.

### 3.3. Calcium Imaging Experiment

Digital calcium imaging was performed as described by Ming *et al.* [34]. Hippocampal neurons were washed three times with 1 μmol/L Fura-2/AM in artificial cerebrospinal fluid (ACSF, containing 140 mM NaCl, 5 mM KCl, 1 mM MgCl_2_, 2 mM CaCl_2_, 10 mM glucose and 10 mM HEPES, pH 7.3) then incubated in the same solution for 30 min at 37 *°*C. In calcium-free experiments, EGTA (100 μM) was substituted for CaCl_2_. Before each experiment, the coverslides were mounted on a chamber positioned on the movable stage of an inverted Olympus IX-70 microscope equipped with a calcium imaging system (TILL Photonics GmbH, Gräfelfing, Germany), and superfused by ACSF for 10 min. Fura-2/AM loaded cells were illuminated at 340 nm for 150 ms and 380 nm for 50 ms at 1-s intervals using a TILL Polychrome monochromator. Fura-2 fluorescence emission was imaged at 510 nm by an intensified cooled charge coupled device (TILL Photonic GmbH) through an X-70 fluor oil immersion lens (Olympus) and a 460 nm long-pass barrier filter. F340/F380 fluorescence ratios were generated by TILLvision 4.0 software. Paired F340/F380 fluorescence ratio images were acquired every second for [Ca^2+^]_i_. The intracellular free calcium concentration is presented as the ratio of the fluorescence signals obtained (340/380 nm). All experiments were repeated at least three times using different batches of cells.

### 3.4. Whole Cell Patch Clamp Recording

The procedure for whole cell patch-clamp recording was described in our previous study [35]. Currents were recorded with whole-cell patch-clamp technique, using an EPC-10 amplifier (HEKA, Lambrecht, Germany) controlled by the Pulse/PulseFit software (HEKA, Southboro, Germany). Data were acquired at a sampling rate of 10 kHz and filtered at 3 kHz. The pipette tips were heat-polished and have resistances of 2–4 MΩ when filled with intracellular buffer. The pipette solution used to record calcium currents consisted of (in mM) CsCl 100, MgCl_2_ 2.0, Na_2_ATP 5.0, egtazic acid 11, HEPES 10, TEA-Cl 20, creatine phosphate 20, and the pH was adjusted to 7.2 with CsOH. The extracellular solution composed of (in mM) NaCl 110, KCl 5.0, CaCl_2_ 5.0, MgCl_2_ 1.0, HEPES 10, Glucose 11, 4-AP 5.0, TEA-Cl 25, TTX 0.5 × 10^−3^ and the pH was adjusted to 7.4 with NaOH. NMDA currents were recorded using electrodes filled with (concentration in mM) cesium gluconate 135, CsCl_5_, CaCl_2_ 5.0, MgCl_2_ 1.0, HEPES 10, Glucose 11, pH 7.2, with Tris base, and the bath solution contained (in mM) NaCl 140, KCl 5, CaCl_2_ 2.5, HEPES 10, Glucose 10, TTX 0.5 × 10^−3^, pH 7.4 with Tris base. 100 μM NMDA was applied to cells for 15 s using a modified Y-tube. Drug actions were measured only after steady-state conditions reached, which were judged by the amplitudes and time courses of currents remaining constant.

### 3.5. Statistical Analysis

The amplitudes of [Ca^2+^]_i_ elevation over the basal were represented as the difference between baseline level [(F340/F380)_B_] and the peak amplitude [(F340/F380)_S_] response to the stimulation, which was shown as [(F340/F380)_S_-F340/F380)_B_]/(F340/F380)_B_. Data are presented as mean ± SEM. Data were analyzed using SPSS 10.0 software. Student’s *t*-test or one-way ANOVA was used to test for significance. Differences were considered significant at *P <* 0.05.

## 4. Conclusions

Results presented here suggested that EGCG triggered a cascade of events: it activated phospholipase C, mobilized intracellular calcium stores, raised the cytosolic Ca^2+^ levels, and inhibited the VGCC and NMDA receptor-mediated Ca^2+^ influxes. Although the mechanisms by which EGCG raised [Ca^2+^]_i_ and inhibited I_HVA_ and I_NMDA_ in cultured hippocampal neurons remain a matter of conjecture, our results may help to understand the neurotoxicity and officinal value of EGCG.
